# Results of Endoscopic Endonasal Dacryocystorhinostomy; Necessity of Teamwork and Patient Selection

**Published:** 2015

**Authors:** Reza ERFANIAN SALIM, Shabahang MOHAMMADI

**Affiliations:** Noor Eye Research Center, Noor Eye Hospital, Tehran, Iran

**Keywords:** Endonasal Dacryocystorhinostomy, Teamwork, Patient Selection

## Abstract

Our aim was to evaluate the clinical results of endoscopic endonasal surgical dacryocystorhinostomy (EES-DCR) as team work by an ophthalmologist and an ear-nose-throat (ENT) surgeon and the appropriate selection of the patients. All candidates for DCR underwent computed tomography (CT) scan of the paranasal sinuses (PNS). Patients who did not want a scar on the medial canthus skin or who had intranasal problems received EES-DCR, which was performed as team work by an ophthalmologist and an ENT surgeon. Surgical success was the resolution of epiphora (i.e., functional success) and free passage of the fluid on irrigation (i.e., anatomical success) by six months after surgery. One hundred twenty-eight patients underwent EES-DCR. Six months after the operation, six patients had surgical failure (three cases of anatomical failure and three cases of functional failure); the success rate was therefore 95.3%. The most common intranasal problems that led to EES-DCR were septal deviation, sinusitis, close proximity of the agger nasi to the lacrimal bone, and concha bullosa; moreover, 15.5% of patients selected EES-DCR for cosmetic reasons. In conclusion, Cooperation between ophthalmologists and ENT surgeons in the preoperative assessment of patients with epiphora before EES-DCR increases its success rate, and it can replace external DCR in some patients.

## INTRODUCTION

After endoscopy for nasal and sinus surgery became widely used (1, 2), this technique was also used for dacryocystorhinostomy (DCR) (3). However, because of their relative mastery of the technique, otorhinolaryngologists tend to use DCR more frequently than ophthalmologists (4). On the other hand, ophthalmologists evaluate patients with epiphora and select patients for DCR surgery, rather than an otorhinolaryngologist (6).

During recent years, because of the widespread use of endoscopic endonasal DCR (EES-DCR) and increased experience and knowledge in this regard, its success rate has increased from approximately 70% to nearly 92.5%, which is equal to external DCR (EX-DCR ) (7). Therefore, this technique could be an alternative to EX-DCR (7).

Based on studies that have attributed DCR failure to intranasal problems (8, 9) or reported the necessity of surgery, along with DCR, for other intranasal problems (6, 10), and with regard to the belief that any attempt to create a reliable connection between the lacrimal sac and the nose increases the odds of the success of DCR (6), this study was conducted as a teamwork project with the participation of an oculoplastic surgery fellow and an otorhinolaryngologist to investigate the results and success rate of EES-DCR in patients who were selected for surgery, based on certain preoperative evaluations.

## METHODS

This prospective nonrandomized clinical trial was conducted in the Oculoplastic and Strabismus Department of Noor Eye Hospital (Tehran, Iran) between April 2012 and March 2014 in accordance with the ethical guidelines of the Noor Eye Hospital Research Center. This study was conducted on patients who had a chief complaint of excess tearing, had attended one of the three oculoplastic clinics of Noor Eye Hospital, and were evaluated by an oculoplastic surgery fellow. For these patients, complete ophthalmological examinations included slit lamp examination, measurement of intraocular pressure (IOP), and fundoscopy with pupil dilation. All patients received irrigation of the lacrimal duct from the upper and lower puncta of the epiphoric eye, unless chronic dacryocystitis was diagnosed by applying pressure on the medial canthus and a pus discharge occurred. The patients with the following criteria were selected for DCR:

1. Patients with chronic dacryocystitis;

2. Patients with primary acquired nasolacrimal duct obstruction (i.e., lack of serum passage from the nasolacrimal duct during irrigation);

3. Patients with epiphora, who had lacrimal duct obstruction, based on nasolacrimal duct (NLD) scintigraphy, despite serum passage with lacrimal duct irrigation; the epiphora disturbed their daily living and there was no other functional cause for their epiphora. These patients were included with a diagnosis of NLD stenosis.

The following patients with lacrimal duct obstruction were removed from analysis, even if they underwent DCR surgery:

- All patients with a previous history of intraocular surgery;
- All patients with a previous history of intranasal surgery;
- All patients with a history of unsuccessful DCR surgery on the affected side;
- All patients with a history of facial and nasal bone fractures;
- All patients with inflammatory diseases of the eye and palpebral disorders that predispose the patient to epiphora;
- All patients with acute dacryocystitis;
- All patients with bilateral lacrimal duct obstruction.

The surgical results of the latter two groups were excluded from analysis for presentation in other papers.

The DCR candidates underwent axial and coronal CT scan of the paranasal sinuses (PNSs) without contrast. They were selected for EES-DCR if CT showed septal deviation to the side of the affected lacrimal duct, sinusitis, concha bullosa on the affected side, and agger nasi close to the lacrimal bone. The ENT consultation was performed with the otorhinolaryngologist, who was in charge of the study. During the consultation, endonasal endoscopy was performed in the ENT clinic, important factors for endonasal surgery were evaluated, and the patient was placed on the EES-DCR surgery waiting list.

Patients with negative findings on the PNS CT scan were provided an opportunity to choose between EES-DCR and EX-DCR, after they were informed about the advantages and disadvantages of each method.

The patients who selected EES-DCR to prevent medial canthus scaring underwent surgery under cosmetic indications, whereas the remaining patients underwent EX-DCR by the oculoplastic surgeon.

**Surgical method**

Endoscopic endonasal DCR (EES-DCR)

The ENT specialist and ophthalmologist were both present during surgery. The type of anesthesia (i.e., general or topical) was not a topic of investigation and was selected based on the preoperative anesthesiology consultation.

A mixture of lidocaine (2%) and epinephrine (1:200,000) was injected into the nasal mucosa in the surgical site in all patients (i.e., topical or general anesthesia). In patients with topical anesthesia, an injection was also administered from the skin side in the medial canthus. A mesh soaked in lidocaine ointment and phenylephrine (0.5%) drops was then placed in the nasal cavity for 15 minutes. If septoplasty was required, the ENT surgeon performed it before DCR.

Through a monitor screen connected to a rigid nasal fibrotic endoscope and by using a periosteum elevator, the primary mucosal incision was formed along the maxillary line in front of the middle turbinate attachment.

The excision of the nasal mucosa was extended to reach behind the posterior crest of the lacrimal bone using a polyp shaver. Sinus surgery and the removal of agger nasi cells or concha bullosa were performed, if required. The lacrimal bone in front of the lacrimal sac fundus was removed with a Kierson or a Medtronic drill (Medtronic, Novi, MI, USA), if required, and the medial wall of the lacrimal sac was entirely excised. The precise location of the new ostium was determined by probes passed through both upper and lower canaliculi. The surgeon carefully produced adequate space in front of the new osteotomy position; therefore, a hemi-turbinectomy was also performed.

After ensuring the lack of an active hemorrhage, silicone tubes were passed through both upper and lower canaliculi and fixed in the nose. In all patients, a mesh soaked in lidocaine, erythromycin, and betamethasone was placed in the nasal cavity. All patients were advised to remain in a semi-seated position and to use cold compress for 48 hours after the surgery and to instill betamethasone and chloramphenicol eye drops in the eye on the operated side every 6 hours for 1 week. The patients also received cephalexin (500 mg) four times daily.

After two days, one-half of the mesh was removed; the remaining mesh was removed after 5 days. After the surgery, ophthalmological examinations were conducted at 1 week, 1 month, and 2 months. At every visit, the new passageway was irrigated with normal saline solution, even with the silicone tube in place.

Periodic intranasal examinations were also scheduled at the same intervals. Intranasal irrigation with normal saline solution was performed and intranasal corticosteroid spray was prescribed. The silicone tube was removed after 8–10 weeks postoperation but the patients were followed up every 2 months for 6 months after the surgery.

In patients who underwent EX-DCR, the standard surgery was performed and silicone tubes were placed. Postoperative care, medications, and follow-up intervals were similar to EES-DCR patients, but the patients were supervised by the ophthalmologist.

Surgical success was defined as resolution of epiphora (i.e., functional success) and free passage of the irrigation fluid (i.e., anatomical success) within 6 months after the surgery. The absence of each item was considered surgical failure.

## RESULTS

Among the DCR surgery candidates, 62 patients underwent EX-DCR. After 6 months follow-up, there were only two patients with anatomical failure. Surgery was successful in the remaining 60 (96.8%) patients. The two patients with anatomical failure underwent EES-DCR. Both patients had some degree of septal deviation on the operated side, which could have been the reason for surgical failure. Septal deviation in these patients was not evident on the preoperative PNS CT images.

On the other hand, 128 patients underwent EES-DCR of whom 49 (38.3%) patients were men and 79 (61.7%) patients were women with a mean age of 50.6 ± 15.66 years (range, 7–83 years). There were only six cases of surgical failure in the 6-month follow-up; therefore, the success rate of EES-DCR was 95.3% in our study.

Among the six failure cases, three patients had functional failure in whom epiphora was resolved by repassing the silicone tube. Three patients with anatomical failure underwent a second EES-DCR. The reason for failure in all three patients was ostial fibrosis. Therefore, with regard to anatomical patency, the success rate was 97.7%.

Among the 128 patients who underwent EES-DCR, 20 patients selected this method for cosmetic reasons (e.g., to avoid scar formation on the face). The remaining patients (i.e., 108 patients) chose this method because of intranasal problems. The most common intranasal problem that led to the selection of EES-DCR was septal deviation, which was the only reason for EES-DCR in 23 (18.1%) patients; it was present with other nasal problems in 58 (51%) patients. Sinusitis was another intranasal problem that was detected alone in 21 (16.5%) patients and with other nasal problems in 51 (46.4%) patients.

The presence of agger nasi air cells close to the lacrimal bone alone or in combination with other problems was the surgical indication for EES-DCR in 7 (5.5%) patients and in 25 (19.5%) patients, respectively. Concha bullosa alone or in combination with other nasal problems was the reason for EES-DCR in 3 (12.4%) patients and 26 (20%) patients, respectively. There were three or more nasal problems in eight of 70 (63%) patients, who had more than one intranasal problem.

## DISCUSSION

The effect of inflammation and intranasal infections has been documented in the pathogenesis of disorders of tear drainage through the nasolacrimal duct into the nose (11), although there have been disagreements in this regard on clinical evaluations. Some studies report that chronic sinusitis in patients who undergo DCR was not more common or more severe than in the normal population (12). However, some investigators have found a higher prevalence of nasal and sinus pathologic conditions in DCR patients (10, 13) in such a way that a considerable number of patients required another intranasal operation in addition to DCR (6, 10). This finding was more common in the re-DCR surgery than in the primary DCR (10, 14). Intranasal pathologies should be corrected before or simultaneously with primary DCR (15).

The lacrimal sac and nasolacrimal duct are surrounded by the bony parts of the lacrimal bone, anterior ethmoid cells, and the frontal process of the maxillary bone (16). The success of DCR depends on the creation of an adequate space in front of the common canaliculi and upper segment of the lacrimal sac (17) (18), which is necessary to prevent adhesion and scarring at the site of rhinostomy (17). For this reason, some intranasal factors such as septal deviation, concha bullosa, middle turbinate hypertrophy, and nasal polyp prepare the ground for surgical failure after DCR surgery because of the restricted space in front of rhinostomy following DCR surgery (8, 9).

The uncinate process has anatomic variations in the nasal cavity and may even completely cover the lacrimal bone area (16). It may therefore need to be removed during DCR surgery (19).

In the normal population, the incidence of the presence of agger nasi cells is more than 80% (20); however, agger nasi cells are close to the lacrimal sac in 55% of patients and should be removed during DCR surgery (16). On the other hand, during the DCR surgery, especially EX-DCR, it is possible to injure the frontal and ethmoid sinus, middle turbinate, and nasal septum, which leads to adhesion after surgery (19).

An ophthalmologist and an otorhinolaryngologist have surgical skill for one end of the tear drainage system (i.e., from the orbit to the nose) (5). For this reason, ophthalmologists still prefer to inj and only 12% of them perform EES-DCR alone (20); however, since the introduction of endoscopic methods in endonasal surgery, the tendency of ENT specialists to perform DCR surgery through the nose has increased (4), and most of them perform the surgery alone. However, most patients are referred to ENT specialists by ophthalmologists because they are responsible for evaluating patients with epiphora and determining the surgical indication of the DCR procedure (5). Therefore, the most important reason for ENT specialists’ failure for EES-DCR surgery is incorrect selection of patients with epiphora because they do not properly investigate causes other than tear duct obstruction (21). On the other hand, intranasal operations that are required during DCR surgery are beyond the knowledge and expertise of most ophthalmologists; therefore, a collaborative surgery helps to locate the place of osteotomy more accurately, evaluate intranasal anatomic variations and pathologic conditions; treat patients more efficiently, and increase the odds of surgical success by decreasing complications (22, 23, 24). For these reasons, teamwork and cooperation between ENT surgeons and ophthalmologists is recommended (24).

The current study was the result of a teamwork project between an oculoplastic surgery fellow and an otorhinolaryngologist with expertise in endoscopic sinus and nasal surgery. The ophthalmologist evaluated patients with epiphora and determined the indication for DCR surgery and the ENT surgeon determined the need for other intranasal operations. Operations were also performed as teamwork. A few patients who underwent EES-DCR selected this surgical method for cosmetic reasons. In patients who underwent EX-DCR for intranasal problems, the most common reasons, in order of frequency, were septal deviation, sinusitis, agger nasi, and concha bullosa. Most patients had more than one problem. Among these patients, there were three cases of anatomic failure and three cases of functional failure.

In general, the success rate of EES-DCR in our study was 95.3%, which was close to the results of other studies. The findings of our study showed that careful evaluation of patients, detection of intranasal problems before the surgery, appropriate selection of the candidates for EES-DCR surgery, and collaborative surgery and teamwork between ophthalmologists and ENT surgeons could increase the success rate of the procedure. Another interesting finding of the study was the low number of patients who selected the EES-DCR method for cosmetic reasons (15.5%).

A limitation of this study was that only patients who had evidence of intranasal problems on CT images (108 of 190 patients) were referred for ENT consultation and none of the patients who underwent EX-DCR received an ENT consultation. It is possible that there were intranasal problems that were not evident on CT images and required endonasal endoscopy in the clinic to be detected. On the other hand, it is possible that if all patients had been referred for ENT consultation, the number of the patients may have been EES-DCR increased. Both patients with EX-DCR failure had septal deviation that was not evident on CT images.

Regardless of the aforementioned advantages of teamwork in EES-DCR surgery, an important disadvantage is the increased number of follow-up visits, as noted by the authors. The patients should be visited by the ophthalmologist and by the ENT specialist for postoperative care. The solution to this problem may be a joint clinic for lacrimal problems and the presence of both specialists in a clinic to visit the patients at the same time. In conclusion, careful evaluation of patients with epiphora and appropriate selection of the patients for EES-DCR surgery and collaborative surgery between and ENT specialist and ophthalmologist increase the success rate of EES-DCR and make this procedure a suitable alternative to EX-DCR in some patients.
